# Leveraging unstructured data to identify hereditary angioedema patients in electronic medical records

**DOI:** 10.1186/s13223-021-00541-6

**Published:** 2021-04-20

**Authors:** Emily S. Brouwer, Emily W. Bratton, Aimee M. Near, Lynn Sanders, Christina D. Mack

**Affiliations:** 1 Takeda Pharmaceutical Company Limited, 300 Shire Way, Lexington, MA USA; 2 IQVIA, Durham, NC USA

**Keywords:** Electronic medical records, Epidemiology, Feasibility study, Hereditary angioedema, Real-world data, Unstructured data

## Abstract

**Background:**

The epidemiologic impact of hereditary angioedema (HAE) is difficult to quantify, due to misclassification in retrospective studies resulting from non-specific diagnostic coding. The aim of this study was to identify cohorts of patients with HAE-1/2 by evaluating structured and unstructured data in a US ambulatory electronic medical record (EMR) database.

**Methods:**

A retrospective feasibility study was performed using the GE Centricity EMR Database (2006–2017). Patients with ≥ 1 diagnosis code for HAE-1/2 (International Classification of Diseases, Ninth Revision, Clinical Modification 277.6 or International Classification of Diseases, Tenth Revision, Clinical Modification D84.1) and/or ≥ 1 physician note regarding HAE-1/2 and ≥ 6 months’ data before and after the earliest code or note (index date) were included. Two mutually exclusive cohorts were created: probable HAE (≥ 2 codes or ≥ 2 notes on separate days) and suspected HAE (only 1 code or note). The impact of manually reviewing physician notes on cohort formation was assessed, and demographic and clinical characteristics of the 2 final cohorts were described.

**Results:**

Initially, 1691 patients were identified: 190 and 1501 in the probable and suspected HAE cohorts, respectively. After physician note review, the confirmed HAE cohort comprised 254 patients and the suspected HAE cohort decreased to 1299 patients; 138 patients were determined not to have HAE and were excluded. The overall false-positive rate for the initial algorithms was 8.2%. Across final cohorts, the median age was 50 years and > 60% of patients were female. HAE-specific prescriptions were identified for 31% and 2% of the confirmed and suspected HAE cohorts, respectively.

**Conclusions:**

Unstructured EMR data can provide valuable information for identifying patients with HAE-1/2. Further research is needed to develop algorithms for more representative HAE cohorts in retrospective studies.

## Background

In the United States, 10% of the population is estimated to be affected by 1 of > 7000 rare diseases [1]. Gaining real-world insights to improve diagnosis rates and inform treatment choices in rare diseases is particularly challenging due to small population sizes, disease complexity, and lack of awareness/expertise within the health care community [2, 3].

Hereditary angioedema type 1/2 (HAE-1/2) is a rare genetic disease that, from a systematic review of studies in European countries, is estimated to impact ~ 1 in 67,000 individuals [4], but much remains unknown about its prevalence, and there are likely many undiagnosed cases. Misdiagnoses and delayed diagnoses are common; data from 1 European registry suggested a median diagnostic delay of 8.5 years, and that 44.3% of eligible patients had ≥ 1 prior misdiagnosis [5, 6].

Symptoms of HAE that overlap with more common disorders, such as allergic angioedema and appendicitis, can make accurate and rapid diagnosis challenging [5, 6]. HAE is characterized by swelling attacks caused by bradykinin-mediated vascular reaction of deep dermal/subcutaneous or mucosal/submucosal tissues [7]. HAE attacks are recurrent and unpredictable in frequency, duration, and location [8, 9]. Severity can range widely; laryngeal swelling poses a fatal threat due to asphyxiation, abdominal attacks are debilitating, and peripheral attacks in hands and/or feet inhibit daily functioning [7]. The epidemiologic impact of HAE is difficult to quantify due to the challenges of diagnosis combined with the necessity of using non-specific diagnosis codes, thus impacting the accuracy of studies that leverage existing data.

Electronic medical records (EMRs) have been adopted widely over the last decade, providing a valuable longitudinal data source to evaluate disease diagnosis, treatment, and quality of care, focused on patients who receive standard of care in real-world settings [10–12]. Data from EMRs have been used extensively to gain insights into patient populations across many diseases in terms of therapeutic product safety, health care and treatment utilization, and guideline adherence, and to establish the epidemiology of diseases [13, 14]. The successful leveraging of EMR data in rare diseases is particularly attractive because of the small numbers of patients and disease experts participating in randomized controlled trials [1], as well as the potential to overcome limitations associated with clinical trial study design, such as slow data collection, short follow-up duration, and underrepresented subgroups [15]. However, the use of EMR databases for investigating HAE can be challenging due to the lack of specific administrative billing codes for this condition, combined with the frequency of delayed diagnosis and/or misdiagnosis.

Furthermore, EMR data may be structured (e.g., diagnosis and procedure codes) or unstructured (i.e., in the form of narrative text from treating physicians) [16]. Although structured EMR data have been used to identify patients in many therapeutic areas, several examples have highlighted the need to investigate unstructured data for both common diseases and rare diseases, such as HAE, to improve the accuracy of identified disease cohorts and to better understand disease characteristics [13, 17–22]. This retrospective database study investigated the feasibility of using structured data coupled with unstructured data in an ambulatory EMR database to identify a real-world cohort of patients with HAE in the United States, describing their demographics, clinical characteristics, and treatment.

## Methods

Patient records were sourced from the GE Centricity EMR Database from IQVIA, from January 1, 2006 to December 31, 2017. This database covers > 33,000 health care providers across 725 institutions and contains 37 million de-identified active patient records (as of May 2017). Because the study utilized existing de-identified patient data, approval from an institutional review board was not required.

Eligible patients had ≥ 1 diagnosis of HAE-1/2, defined using International Classification of Diseases, Ninth Revision, Clinical Modification (ICD-9-CM) code 277.6 or International Classification of Diseases, Tenth Revision, Clinical Modification (ICD-10-CM) code D84.1 and/or mention of HAE-1/2 diagnosis in unstructured physician notes. The index date was the date of the first diagnosis code or first mention of HAE in physician notes. Patients were required to have ≥ 6 months of available look-back data before the index date (baseline period), defined as ≥ 1 EMR visit during that time, and ≥ 6 months of available data after the index date (follow-up period). The duration of follow-up varied, with patients being followed until whichever of these occurred first: no visit within a 6-month period, death, or the end of the data window. Patients with incomplete records (e.g., missing age or sex) or with other data quality issues were excluded.

Two mutually exclusive study cohorts were created (Table 1). The first cohort was labeled the “probable HAE cohort” and was defined as having either ≥ 2 diagnosis codes or ≥ 2 mentions of HAE in physician notes, or ≥ 1 code plus ≥ 1 HAE mention in notes; multiple diagnosis codes and/or notes were required to occur on separate days. The second cohort, the “suspected HAE cohort,” was defined as having either only 1 diagnosis code or only 1 mention of HAE in physician notes.

**Table 1:** Algorithm definitions for initial study cohorts

Study cohort	Definition
Probable HAE	≥ 2 ICD-9-CM/ICD-10-CM diagnosis codes^a^ on separate days and no mention of HAE in physician notes **OR** ≥ 2 mentions of HAE in physician notes on separate days and no diagnosis code^a^ **OR** ≥ 1 diagnosis code^a^ and ≥ 1 mention of HAE in physician notes
Suspected HAE	Only 1 diagnosis code^a^ **OR** Only 1 mention of HAE in physician notes and no diagnosis code^a^

^a^ICD-9-CM 277.6 or ICD-10-CM D84.1

*HAE* hereditary angioedema, *ICD-9-CM* International Classification of Diseases, Ninth Revision, Clinical Modification, *ICD-10-CM* International Classification of Diseases, Tenth Revision, Clinical Modification

After inclusion into the 2 initial study cohorts, physician notes of patients selected into a cohort by ≥ 1 note were manually reviewed by two epidemiologists (AMN and Sara Waugh, IQVIA), with a third reviewer (EWB) available in case of disagreement. The impact of this refinement of diagnostic criteria was quantified, with manual review of unstructured data determining the final classification of “confirmed” or “suspected” HAE, and including the creation of a third cohort of patients for whom review of physician notes indicated that HAE was not diagnosed; these patients were removed from the study. Unstructured data that reviewers used to assign patients to the “confirmed HAE cohort” included: diagnosis codes ICD-9-CM 277.6 or ICD-10-CM D84.1; “hereditary angioedema” or “HAE”; C1 or C4 levels that indicate HAE-1/2; mention of medication(s) specifically used to treat HAE (including C1 inhibitor, icatibant, ecallantide, and androgens); and language such as “confirmed,” “patient has,” “patient diagnosed with,” “history of,” “likely,” or “treated for.” A patient was assigned to the final “suspected HAE cohort” if physician notes mentioned any of the following: symptoms in the context of acute respiratory, dermatological, or gastrointestinal events (episodes or attacks) known to be associated with having HAE; and language such as “pending work-up for,” “being evaluated for,” “testing sent/ordered to rule out HAE,” or “consider HAE diagnosis.” A patient was removed from the cohort if physician notes included the presence of only a family history or family member with HAE, C1 or C4 levels that did not indicate HAE-1/2, or language such as “unlikely to have” or “does not have.”

Physician notes were considered decisive in terms of diagnostic status; for example, if a patient was selected into the suspected HAE cohort based on the inclusion criteria, but the physician notes confirmed a diagnosis of HAE-1/2, the patient was moved to a final confirmed HAE cohort. The false-positive rate with respect to the use of diagnosis codes and/or the physician note definition for evidence of confirmed patients with HAE was calculated as the number of patients removed from the probable or suspected HAE cohorts, divided by the total number of patients originally identified.

The final 2 study cohorts, after removal of patients considered not to have HAE, were described in terms of patient characteristics at baseline and during the follow-up period. Variables analyzed included demographic characteristics, insurance information, medical diagnoses, clinical characteristics, diagnostic tests and results, procedures, and prescription orders related to the treatment of HAE. Disease characteristics described include 15 comorbidities common to patients with HAE; prescriptions for HAE-specific treatment (C1 inhibitors, ecallantide, icatibant, and androgens); and evidence of HAE attacks through either pre-specified diagnosis/procedure codes (for swelling/angioedema, abdominal pain, asphyxiation, laryngoscopy, or esophagogastroduodenoscopy), along with the potential attack location (gastrointestinal, laryngeal/respiratory, or subcutaneous), or mention in physician notes of “HAE” (or “hereditary angioedema”) plus either the potential attack location (consistent with HAE attack manifestations) or ≥ 1 of the words, “episode,” “attack,” “edema,” “swelling,” “anaphylaxis,” “anaphylactic,” or “event.” The number and percentage of patients with documented evidence of ≥ 1 HAE attack and the number of attacks per patient per month (PPPM) were reported.

## Results

### Study population and cohort formation

**Fig. 1:**
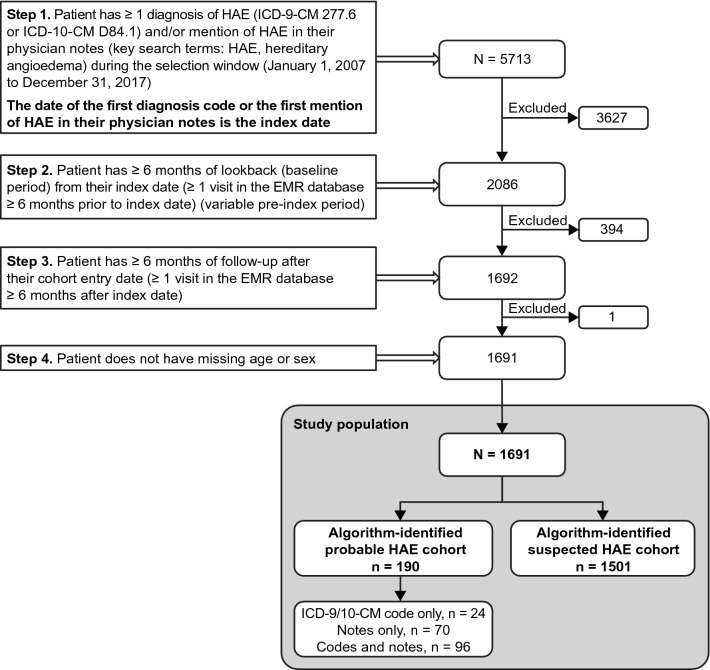
Patient selection. *EMR* electronic medical record, *HAE* hereditary angioedema, *ICD-9-CM* International Classification of Diseases, Ninth Revision, Clinical Modification, *ICD-10-CM* International Classification of Diseases, Tenth Revision, Clinical Modification

A total of 1691 patients met the eligibility criteria and comprised the study population (Fig. 1). Using the algorithms defined in Table 1, 190 patients were assigned to the initial probable HAE cohort and 1501 patients to the initial suspected HAE cohort. Physician notes of patients with ≥ 1 mention of HAE were then reviewed, resulting in patient numbers of 254 in the final confirmed HAE cohort and 1299 in the final suspected HAE cohort.

**Fig. 2:**
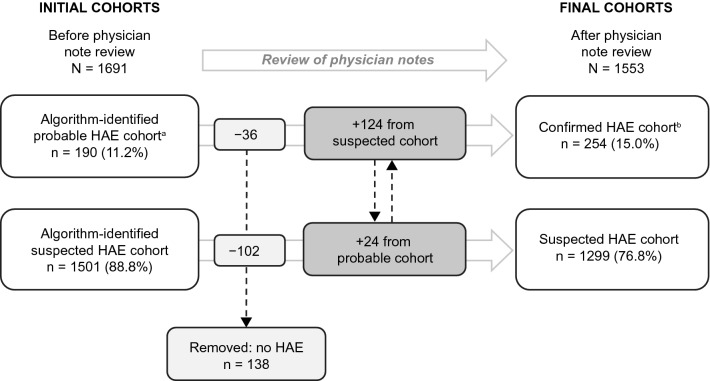
Impact of physician note review on composition of final study cohorts. ^a^Diagnosis codes only, n = 24; notes only, n = 70; codes and notes, n = 96. ^b^Diagnosis codes only, n = 24; notes only, n = 22; codes and notes, n = 84; 1 note mention, n = 124. *HAE* hereditary angioedema

A total of 138 patients were removed from the 2 initial cohorts because HAE-1/2 diagnosis was mentioned as a rule-out diagnosis in the physician notes, or only a family history of HAE was mentioned (Fig. 2). The overall false-positive rate for the initial algorithms was 8.2%.

Among the probable HAE cohort, 24 patients had ≥ 2 diagnosis codes, 70 had ≥ 2 mentions of HAE in physician notes, and 96 had a mixture of ≥ 1 diagnosis code and ≥ 1 physician note. After review of physician notes, 36 patients were excluded from the study, 24 were moved from the probable cohort to the suspected HAE cohort, and 124 were moved from the suspected HAE cohort to the probable cohort to increase the size of the final confirmed HAE cohort (Fig. 2).

### Baseline demographic and clinical characteristics

The final study cohorts were described in terms of baseline demographic and clinical characteristics (Tables 2, 3). The median age at first diagnosis code or physician note was 50 years for both cohorts. Female patients comprised 62.2% of the confirmed HAE cohort and 63.3% of the suspected HAE cohort. Approximately half of patients in both cohorts were commercially insured. Physician specialties within the GE Centricity EMR Database are listed in Table 4, with the majority of patients receiving their diagnosis by a primary care physician.

**Table 2:** Baseline demographic characteristics of the final study cohorts

Characteristic	Confirmed HAE (n = 254)	Suspected HAE (n = 1299)
Age (years)		
Mean (SD)	46.3 (20.6)	46.2 (21.3)
Median (IQR)	50 (29–61)	50 (31–62)
Range	1–90	0–97
Age category (years), n (%)		
5–14	14 (5.5)	98 (7.5)
15–24	29 (11.4)	104 (8.0)
25–34	31 (12.2)	118 (9.1)
35–44	21 (8.3)	173 (13.3)
45–54	52 (20.5)	224 (17.2)
55–64	50 (19.7)	260 (20.0)
65–74	36 (14.2)	189 (14.5)
≥ 75	16 (6.3)	86 (6.6)
Sex, n (%)		
Male	96 (37.8)	477 (36.7)
Female	158 (62.2)	822 (63.3)
Race/ethnicity, n (%)		
White	195 (76.8)	932 (71.7)
Black	21 (8.3)	162 (12.5)
Asian	1 (0.4)	14 (1.1)
Native American or Pacific Islander	2 (0.8)	10 (0.8)
Unknown	35 (13.8)	181 (13.9)
Geographic region, n (%)		
Northeast	65 (25.6)	279 (21.5)
Midwest	57 (22.4)	198 (15.2)
South	90 (35.4)	613 (47.2)
West	39 (15.4)	194 (14.9)
Unknown	3 (1.2)	15 (1.2)
Payer type, n (%)		
Commercial	119 (46.9)	662 (51.0)
Medicaid	10 (3.9)	45 (3.5)
Medicare	28 (11.0)	162 (12.5)
TRICARE (military)	5 (2.0)	20 (1.5)
Self-insured	15 (5.9)	30 (2.3)
Other/unknown	77 (30.3)	380 (29.3)
Year of index date, n (%)		
2007	15 (5.9)	56 (4.3)
2008	18 (7.1)	103 (7.9)
2009	20 (7.9)	118 (9.1)
2010	23 (9.1)	88 (6.8)
2011	18 (7.1)	104 (8.0)
2012	28 (11.0)	119 (9.2)
2013	18 (7.1)	98 (7.5)
2014	25 (9.8)	129 (9.9)
2015	27 (10.6)	157 (12.1)
2016	48 (18.9)	239 (18.4)
2017	14 (5.5)	88 (6.8)

*HAE* hereditary angioedema, *IQR* interquartile range, *SD* standard deviation

**Table 3:** Baseline clinical characteristics of the final study cohorts

Characteristic	Confirmed HAE (n = 254)	Suspected HAE (n = 1299)
Comorbidity, n (%)		
Allergy/anaphylaxis	61 (24.0)	498 (38.3)
Hypertension	50 (19.7)	276 (21.2)
Anxiety	28 (11.0)	125 (9.6)
Arthritis	26 (10.2)	94 (7.2)
Depression	22 (8.7)	116 (8.9)
Diabetes	22 (8.7)	102 (7.9)
Urticaria	21 (8.3)	164 (12.6)
Obesity	20 (7.9)	149 (11.5)
Anemia	17 (6.7)	118 (9.1)
Hypothyroidism	17 (6.7)	73 (5.6)
COPD	9 (3.5)	103 (7.9)
Fluid and electrolyte disorders	8 (3.1)	57 (4.4)
Liver disease	3 (1.2)	36 (2.8)
Pruritus	3 (1.2)	37 (2.8)
Hypotension	2 (0.8)	18 (1.4)
Prescriptions for HAE-specific medication		
Patients with ≥ 1 prescription, n (%)	23 (9.1)	13 (1.0)
Prescriptions PPPM		
Mean (SD)	0.10 (0.11)	0.10 (0.08)
Median (IQR)	0.08 (0.03–0.13)	0.05 (0.04–0.14)
Type of treatment, n (%)		
C1 inhibitor	8 (3.1)	2 (0.2)
Ecallantide	0	0
Icatibant	4 (1.6)	1 (0.1)
Androgen	15 (5.9)	11 (0.8)
HAE attack diagnosis and/or procedure codes		
Patients with evidence of ≥ 1 HAE attack, n (%)	115 (45.3)	683 (52.6)
HAE attacks PPPM		
Mean (SD)	0.03 (0.05)	0.04 (0.04)
Median (IQR)	0.02 (0.00–0.04)	0.02 (0.01–0.05)

*COPD* chronic obstructive pulmonary disease, *HAE* hereditary angioedema, *IQR* interquartile range, *PPPM* per patient per month, *SD* standard deviation

**Table 4:** Physician specialties within the GE Centricity EMR Database

Physician specialty, n (%)	Confirmed HAE n = 254	Suspected HAE n = 1299	Removed n = 138
Primary Care	161 (63.4)	786 (60.5)	76 (55.1)
Allergy/Immunology	21 (8.3)	124 (9.5)	12 (8.7)
Dermatology	0	0	0
Gastroenterology	0	0	0
Hematology-Oncology	0	0	0
Obstetrics-Gynecology	2 (0.8)	17 (1.3)	2 (1.4)
Pulmonology	0	0	0
Rheumatology	0	0	0
Other/Unknown	70 (27.6)	372 (28.6)	48 (34.8)

*EMR* electronic medical record*, HAE* hereditary angioedema

Respectively, the mean (standard deviation [SD]) and median (interquartile range) number of office visits PPPM during the baseline period were 0.7 (0.7) and 0.5 (0.2–0.9) for the confirmed HAE cohort and 0.7 (0.7) and 0.5 (0.3–0.9) for the suspected HAE cohort.

The most frequent comorbidities across both cohorts were allergy/anaphylaxis (24.0% in the confirmed HAE cohort and 38.3% in the suspected HAE cohort) followed by hypertension (19.7% and 21.2%, respectively). The proportion of patients with ≥ 1 prescription for HAE-specific medications was low (9.1% and 1.0%, respectively). The most frequently prescribed treatments indicated only for HAE were C1 inhibitors (3.1% and 0.2%, respectively); androgens were used by 5.9% and 0.8% of patients, respectively. Diagnosis and/or procedure codes providing evidence for HAE attacks during baseline were reported for 45.3% of the confirmed HAE cohort and 52.6% of the suspected HAE cohort, and indicated a mean (SD) number of HAE attacks PPPM of 0.03 (0.05) and 0.04 (0.04), respectively.

### Clinical characteristics during the follow-up period

The mean (SD) duration of follow-up was 50.0 (34.3) months in the confirmed HAE cohort and 45.9 (33.1) months in the suspected HAE cohort (Table 5). The proportion of patients with ≥ 1 prescription for HAE-specific medication was substantially higher in the confirmed HAE cohort (31.1%) compared with the suspected HAE cohort (2.3%). C1 inhibitors were recorded in 17.7% of the confirmed HAE cohort and 0.4% in the suspected HAE cohort, and androgens in 13.8% and 1.7%, respectively. Evidence of ≥ 1 HAE attack during follow-up was identified for 41.7% of the confirmed HAE cohort and 29.9% of the suspected HAE cohort, with a mean (SD) number of HAE attacks PPPM of 0.05 (0.09) and 0.03 (0.07), respectively.

**Table 5:** Clinical characteristics during the follow-up period

Characteristic	Confirmed HAE (n = 254)	Suspected HAE (n = 1299)
Duration of follow-up period (months)		
Mean (SD)	50.0 (34.3)	45.9 (33.1)
Median (IQR)	45.7 (19.4–72.0)	37.8 (16.9–70.5)
Number of visits during the follow-up period		
Mean (SD)	1.14 (1.10)	1.02 (0.91)
Median (IQR)	0.77 (0.40–1.48)	0.75 (0.34–1.46)
Prescriptions for HAE-specific medication		
Patients with ≥ 1 prescription, n (%)	79 (31.1)	30 (2.3)
Prescriptions PPPM		
Mean (SD)	0.18 (0.18)	0.13 (0.23)
Median (IQR)	0.10 (0.05–0.29)	0.07 (0.03–0.11)
Type of treatment, n (%)		
C1 inhibitor	45 (17.7)	5 (0.4)
Ecallantide	11 (4.3)	3 (0.2)
Icatibant	26 (10.2)	4 (0.3)
Androgen	35 (13.8)	22 (1.7)
HAE attack diagnosis and/or procedure codes		
Patients with evidence of ≥ 1 HAE attack, n (%)	106 (41.7)	389 (29.9)
HAE attacks PPPM		
Mean (SD)	0.05 (0.09)	0.03 (0.07)
Median (IQR)	0.02 (0.00–0.06)	0.00 (0.00–0.03)

*HAE* hereditary angioedema, *IQR* interquartile range, *PPPM* per patient per month, *SD* standard deviation

## Discussion

To the best of our knowledge, this is the first study to quantify the impact of using both structured and unstructured data from an EMR database to identify and assess a real-world cohort of patients with HAE-1/2. Given that there are no specific diagnosis/procedure codes for HAE-1/2 diagnosis or HAE attacks, several prior claims-based studies have utilized the most commonly used diagnostic code, ICD-9-CM 277.6, either alone or in conjunction with HAE-specific medication claims, to identify patients with HAE-1/2 [23–26]. Although this code, along with ICD-10-CM D84.1, is not typically used for other conditions, the false-positive rate of 8.2% in this study with 138 removed patients reflects the need for caution when identifying patients with HAE-1/2 using only diagnosis codes.

The proportions of patients in our study with prescriptions for HAE-specific medication were higher in the confirmed HAE cohort than in the suspected HAE cohort at baseline and during the follow-up period, highlighting the appropriateness of the cohort definitions. However, only treatments prescribed during the follow-up period of the study were retrieved from the medical records, and patients may have had treatment prescriptions available to them outside of this period. For example, it is recommended that patients diagnosed with HAE have access to on-demand treatment and sufficient medication for 2 acute attacks, and many patients also use long-term prophylaxis [7]. Therefore, the true proportion of patients receiving prescriptions for HAE-indicated medication may have been underrepresented in the study. The relatively low proportion (31.1%) of patients in the confirmed HAE cohort with ≥ 1 prescription during the 6-month follow-up period may have also resulted from patients obtaining confirmed or suspected HAE status from a general provider registered in the database followed by treatment from a specialist provider not registered in the database, resulting in the omission of received treatments in their EMR.

Further studies could investigate the inclusion of prescriptions for HAE-specific medications into the algorithm to increase sensitivity, as this approach may identify patients without a diagnosis code but with evidence of medication for HAE attacks. Nonetheless, the current findings show that review of available physician notes in EMRs provides valuable information to supplement codified fields and mitigate the risk of misclassification of patients with HAE in retrospective studies, although caution must be taken when outlining search terms for the unstructured note mining.

Randomized controlled trials often have narrow inclusion criteria and protocol-directed care that differs from routine clinical care, and there has been increasing interest in the use of real-world evidence to supplement clinical trial data in order to better reflect patient behavior and disease management in uncontrolled care settings [27, 28]. If drug development programs in rare diseases are to successfully utilize retrospective data such as medical records, as advised by the US Food and Drug Administration in draft guidance published in February 2019 [29], then harnessing unstructured data through a systematic and validated approach will improve confidence in the studies’ findings and subsequent recommendations.

The importance of reviewing unstructured data in an EMR database has been demonstrated in several diseases. Earlier diagnosis of patients with chronic diseases such as multiple sclerosis and celiac disease was facilitated in the absence of diagnostic code data [30, 31], and patients with asthma experiencing allergic bronchopulmonary aspergillosis as a disease exacerbation were accurately identified despite the lack of a specific code [32]. Additionally, 2 studies that aimed to identify patients with either congenital or acquired hemophilia found potentially high numbers of false-positive identifications when using diagnostic codes alone [33, 34]. The current study further adds to a body of evidence illustrating the value of using unstructured data, and it is the first to demonstrate utility in HAE, a rare and debilitating disease for which more efficient diagnosis and effective management are needed.

The development of a specific and sensitive algorithm to improve diagnosis rates and lessen delays could have a substantial impact on reducing patient burden and improving quality of care in HAE. The use of unstructured EMR data and natural language processing has been extremely informative in epidemiological and pharmacoepidemiological investigations in other therapy areas [19–21, 32, 35, 36], and provides a unique opportunity for novel insights into the HAE population. Further studies are needed to optimize the search terms used in the current study in order to accurately identify evidence of HAE attacks in physician notes. Given that proportions of patients with evidence of ≥ 1 HAE attack were low and comparable across all cohorts, including the 138 removed patients (results not shown), refining these criteria will be important for increasing the specificity of the final algorithm. The refined methodology could be generalizable to other difficult-to-diagnose illnesses such as fibromyalgia, but may be less applicable to disorders that utilize imaging, biomarkers, or other forms of validated biological testing to clinically verify diagnoses.

This study is subject to limitations inherent in retrospective database studies, where data are not collected for research purposes. Missing data/incomplete records can be common, and coding errors are possible, affecting data quality [37]. The database does not include procedures occurring in a hospital setting; any HAE attacks that led to hospitalization or procedures conducted within a hospital were not captured. EMR data are biased toward more sick individuals who may be patients with higher health care resource utilization [38]. Because of the intermittent nature of HAE attacks, patients who have been diagnosed with HAE but do not have frequent attacks, do not treat mild attacks, or who have controlled symptoms may not have been adequately captured if they were not visiting health care providers within the 6-month follow-up period. Whereas primary care providers are predominant in the GE Centricity EMR Database, patients with HAE-1/2 may be more likely to seek care from a specialist such as an allergist or immunologist [39]. Patients may have received a confirmed or suspected diagnosis from a registered primary care provider but received subsequent treatment from a specialist who was not registered in the database (resulting in treatments not being captured in their EMR). This increases the potential for patients receiving specialist treatment to be missed, and it limits the accurate follow-up of HAE prescribing patterns if a patient leaves their general provider after diagnosis and is routinely seen by a specialist outside of the EMR system for the clinical management of their disease. Finally, because patients with HAE-1/2 often have a long diagnostic journey, those who are not yet diagnosed or have been misdiagnosed with other disorders would not be identified by our algorithm. Further studies that are able to use the identification of correctly diagnosed patients and their health care history may allow for the development of algorithms that can facilitate the earlier diagnosis of patients with HAE.

## Conclusions

Although diagnosis codes have been used to define real-world cohorts of patients with a range of conditions, our findings suggest that there is a risk of underrepresentation and misclassification among patients with HAE-1/2 when relying solely on diagnosis codes. A cohort of 190 patients with HAE-1/2 identified through EMR data was expanded to 254 patients following review of physician notes, highlighting the need to analyze the unstructured data provided in addition to structured data such as diagnostic codes and treatment-based algorithms. Primary data collection through a prospective study may be required to elucidate the patterns and severity of HAE attacks and the impact of long-term prophylactic treatments on patients’ health-related quality of life. Further studies are warranted to identify and validate algorithms that can provide sensitivity and specificity in observational studies that use secondary data, which would allow more rapid and confident data collection, and ultimately could improve our understanding of the epidemiological impact of HAE-1/2.

## Data Availability

All data generated or analyzed during this study are included in this published article.

## Abbreviations

EMR: Electronic medical record
HAE: Hereditary angioedema
ICD-9-CM: International Classification of Diseases, Ninth Revision, Clinical Modification
ICD-10-CM: International Classification of Diseases, Tenth Revision, Clinical Modification
PPPM: Per patient per month; SD: standard deviation
